# Prediction of Nocturnal Hypoglycemia in Adults with Type 1 Diabetes under Multiple Daily Injections Using Continuous Glucose Monitoring and Physical Activity Monitor

**DOI:** 10.3390/s20061705

**Published:** 2020-03-19

**Authors:** Arthur Bertachi, Clara Viñals, Lyvia Biagi, Ivan Contreras, Josep Vehí, Ignacio Conget, Marga Giménez

**Affiliations:** 1 Institute of Informatics and Applications, University of Girona, 17003 Girona, Spain; abertachi@utfpr.edu.br (A.B.); lyviar@utfpr.edu.br (L.B.); ivancontreras@udg.edu (I.C.); 2 Federal University of Technology—Paraná (UTFPR), Guarapuava 85053-525, Brazil; 3 Diabetes Unit, Endocrinology and Nutrition Dpt. Hospital Clínic de Barcelona, 08036 Barcelona, Spain; vinals@clinic.cat (C.V.); iconget@clinic.cat (I.C.); gimenez@clinic.cat (M.G.); 4 Centro de Investigación Biomédica en Red de Diabetes y Enfermedades Metabólicas Asociadas (CIBERDEM), 08036 Barcelona, Spain

**Keywords:** artificial neural network, hypoglycemia, machine learning, support vector machine, type 1 diabetes, multiple daily injections, continuous glucose monitoring

## Abstract

(1) Background: nocturnal hypoglycemia (NH) is one of the most challenging side effects of multiple doses of insulin (MDI) therapy in type 1 diabetes (T1D). This work aimed to investigate the feasibility of a machine-learning-based prediction model to anticipate NH in T1D patients on MDI. (2) Methods: ten T1D adults were studied during 12 weeks. Information regarding T1D management, continuous glucose monitoring (CGM), and from a physical activity tracker were obtained under free-living conditions at home. Supervised machine-learning algorithms were applied to the data, and prediction models were created to forecast the occurrence of NH. Individualized prediction models were generated using multilayer perceptron (MLP) and a support vector machine (SVM). (3) Results: population outcomes indicated that more than 70% of the NH may be avoided with the proposed methodology. The predictions performed by the SVM achieved the best population outcomes, with a sensitivity and specificity of 78.75% and 82.15%, respectively. (4) Conclusions: our study supports the feasibility of using ML techniques to address the prediction of nocturnal hypoglycemia in the daily life of patients with T1D on MDI, using CGM and a physical activity tracker.

## 1. Introduction

Hypoglycemia is the most common side effect of insulin therapy in type 1 diabetes (T1D) and its frequency increases with tight glucose control. It is associated with a range of morbidities, including cardiovascular events and even death due to arrhythmias [1,2]. Almost every aspect of daily life can be influenced by hypoglycemia (e.g., driving, working, recreational activities), which becomes a great burden for individuals [3]. The fear of hypoglycemia may cause some patients to deliberately maintain undesirable hyperglycemia to minimize the risk and severity of further episodes precluding the benefits of tight glycemic control [4,5]. In addition to this, repeated episodes of hypoglycemia induce so-called impaired awareness hypoglycemic (IAH) syndrome, which can lead to severe episodes [6].

More than 50% of severe hypoglycemic episodes occur during the sleep and this is an additional challenge for T1D management [7]. The frequency of nocturnal hypoglycemia (NH), defined as episodes occurring while people are asleep at night, is difficult to ascertain using capillary blood glucose for obvious reasons, although the recent use of continuous glucose monitoring (CGM) revealed that it is even more common than previously suspected, it can last for hours, and it is commonly asymptomatic (70%–80%) because during the night the counter-regulatory responses are significantly blunted [8,9]. The risk factors that predispose an individual to NH are previous hypoglycemia episodes, low A1c, IAH, and increasing duration of T1D, among others. An excessive/wrong insulin dose, inadequate carbohydrate ingestion, alcohol consumption, and previous physical activity are within the circumstances contributing to the development of NH [10,11,12,13].

Continuous subcutaneous insulin infusion (CSII) therapy, CGM, and a combination of both (with the use of a sensor augmented pump (SAP)) have significantly reduced the frequency of hypoglycemia (including NH) [14,15,16,17,18,19]. However, those aforementioned therapies are not suitable for the vast majority of individuals with T1D, who continue to use multiple daily injections of insulin as regular treatment.

As a result of the great socioeconomic impact caused by diabetes mellitus worldwide and the advancement of technology, a large volume of diabetes-related data is available either for physicians to tailor patients’ therapy or for researchers to apply machine-learning (ML) and data-mining techniques. A recent review [20] noted that an impressive growth of articles including the terms “diabetes” and “artificial intelligence” has been observed over the last decade. ML utilization has a wide range of applications in the context of diabetes management. One application of ML methods has focused on the continuous prediction of blood glucose concentration, especially after the introduction of CGM. From a clinical perspective, having a continuous predictor can guide patient decisions regarding the actions required to avoid undesirable hypo- or hyperglycemia; numerous publications are focused on this subject [21,22,23,24]. In the review of blood glucose prediction strategies conducted by Oviedo and colleagues [25], 87% of the publications were focused on the continuous prediction of blood glucose concentration, and only 13% were intended to predict the occurrence of adverse glycemic events.

Very few studies addressed the prediction of hypoglycemic events as a classification problem. Reddy et al. [26] applied ML techniques to predict the occurrence of a hypoglycemic episode while adults with T1D were performing aerobic exercise. Oviedo et al. [27] considered support vector machines (SVM) to predict postprandial hypoglycemia using retrospective data from 10 adults with T1D under SAP therapy. Regarding NH, Tkachenko et al. [28] applied a linear combination to aggregate several heuristic classifiers to forecast such adverse event. However, in this study, only retrospective data from blood glucose concentration was used as input features for their predictors, while it is well established that other variables influence glycemic concentration during the night.

T1D patients using MDI therapy are more exposed to NH than SAP users [29,30], thus more effort should be directed towards this group. The present work evaluates the feasibility of machine-learning-based prediction models to forecast the occurrence of NH, considering data related with T1D management under MDI therapy and also physical activity tracking.

## 2. Materials and Methods

### 2.1. Study Protocol and Subjects

A longitudinal, prospective, interventional, and open label study was conducted at a tertiary endocrinology and nutrition department. It was approved by the local ethical committee and a written informed consent was obtained from each participant. The study was registered under ClinicalTrials.gov (NCT03711656). Patients enrolled in the study met the following inclusion criteria: aged > 18 years, with T1D for at least 5 years, treated with multiple doses of insulin (MDI), using a rapid acting insulin analogue such as prandial insulin and a basal insulin analogue, with HbA1c within 6.5% and 9.5%, practicing carbohydrate (CH) counting, able to understand and follow the instructions of the study including the use of an intermittently scanned continuous glucose monitoring (CGM) system and to perform > 4 self-monitoring blood glucose measurements (SMBG) per day. In addition, they fulfilled the following: >4 hypoglycemia/week (<3.9 mmol/L (70 mg/dL), including day and night), in the last 2 weeks and/or one severe hypoglycemia (needing third party assistance) during the last year and/or impaired awareness hypoglycemia (IAH, Clarke Test > 3). Exclusion criteria included: serious illness that may impair study participation, pregnancy or breastfeeding, history of drug use or alcohol abuse, and the use of an experimental drug or device during the past 30 days.

The study was designed to monitor patients during 12 weeks at home under free-living conditions, and intended to collect data from the daily activities of the patients. During this period, every patient used the CGM FreeStyle Libre (FSL) system (Abbott Diabetes Care, Alameda, CA, USA) to monitor interstitial glucose concentration and the Fitbit Alta HR wristband (Fitbit, Inc., San Francisco, CA, USA) to obtain the data related with physical activity and sleeping periods.

In the initiation visit, patients received FSL sensors, the reader device with its specific blood glucose test strips, and the Fitbit Alta HR wristband. Participants were trained on how to use the devices and on how to upload the data to the respective web servers of each manufacturer. Each patient received between 6 and 10 CGM-sensors with a lifespan of 2 weeks per unit. Patients were instructed to manually enter the information of every insulin dose (rapid-acting and long-acting), as well as the estimation of CH content for every meal consumed during the study, into the reader device. Additionally, patients were asked to wear the wristband as much as possible during the study (including sleeping), removing it just for showering, charging, and practicing water sports. The Fitbit was synchronized with the patient’s mobile phone through a Bluetooth connection.

### 2.2. Data Processing and Feature Engineering

**Figure 1:**
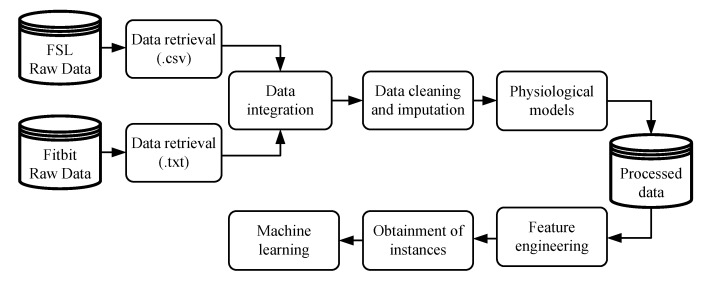
Methodology applied to prepare the data from FreeStyle Libre (FSL) and Fitbit for the application of machine-learning algorithms.

The development of individualized models was carried out through several stages. Figure 1 illustrates the methodology considered in the initial phase, whereby raw data must be prepared to be applied in ML algorithms. The variables collected from the FSL, with their respective timestamps, were interstitial glucose concentration, meals, insulin doses, and SMBG values. The variables collected by the Fitbit, also with their respective timestamps, were heart rate signal, steps performed, estimation of calories burned, and sleeping period.

**Figure 2:**
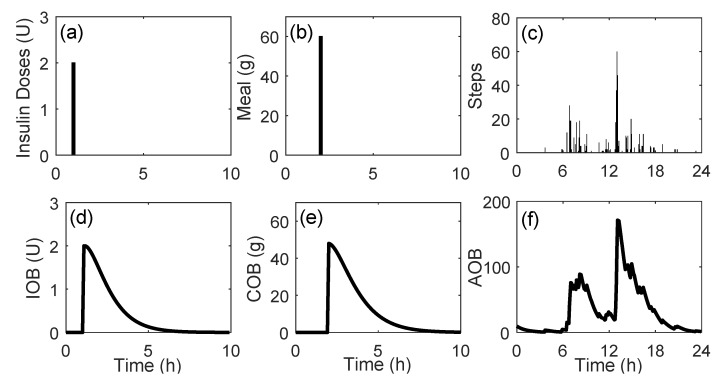
Utilization of the physiological models to generate features. (**a**–**c**) represent a single insulin dose, a single meal consumed, and the steps performed over a single day, respectively. (**d**–**f**) are the insulin on board (IOB), carbohydrate on board (COB), and activity on board (AOB) obtained by the physiological models.

After data integration, the cleaning and imputation procedures were applied. The imputation of missing data was performed in a straightforward way: any gap in interstitial glucose concentration data lower or equal to 120 min was imputed through linear interpolation. In cases of gaps larger than 120 min, no imputation was performed. Finally, a sampling period of 5 min was considered for all the data, reducing the number of samples from a single day to 288. Different physiological models were applied to the data to obtain a representation of the effects of fast-acting insulin doses, announced meals, and steps performed over the day. First, the insulin on board (IOB) model [31] was applied to fast-acting insulin doses. Second, the carbohydrate (CH) on board (COB) model [32] was applied in all the meals stored in the dataset. The COB is conceptually similar to IOB. It represents the amount of CH that has been consumed but that still has not appeared in plasma. Lastly, the effect of physical activity is represented by the activity on board (AOB) model [33], which represents the accumulated effects of physical activity in the body. The signals generated by the physiological models are illustrated in Figure 2.

Once the data were properly manipulated, several time-domain features could be created to be used as attributes for the ML algorithms. A total of 29 features were hand-crafted from the 6 h of data prior to the start of the patient’s sleep period, the period in which the Fitbit informed that patients were sleeping (considering the time instant obtained by the wristband). The features aimed to provide the necessary information to predict NH: one feature related with COB, one feature related IOB, two features related with physical activity (AOB and estimation of calories burned), and the remaining are related with the CGM signal. Class labeling was performed 6 h after the beginning of sleep considering the following: if any interstitial glucose concentration reading was lower than 3.9 mmol/L (70 mg/dL), it was considered an event. Such an instance was labeled as Class 1 (night with hypoglycemia). Otherwise, Class 0 (night without hypoglycemia) was assigned.

### 2.3. Performance Metrics

The performance of the generated classifiers was evaluated by a metric which combines the sensitivity (*SN*) and the specificity (*SP*), computed by Equation (1). The *SN*, or true positive rate, measures the proportion of actual positives that are correctly identified as such, and the *SP*, or true negative rate, measures the proportion of actual negatives that are correctly identified as such. The accuracy of a classifier represents the proportion of classifications performed correctly among all the examples. However, the analysis of a single metric among those presented above may fail to evaluate the performance of a classifier. For example, in the present context, a sensitivity of 100% indicates that every night with hypoglycemia was correctly predicted, but it does not show how many nights without hypoglycemia were wrongly classified, i.e., it does not take into account false positives. An alternative way to evaluate the efficacy of a classifier is to combine some of the previous metrics into a single measure. Equation (1) also defines the Gmean, which considers both SN and SP, giving them the same relevance for classification purposes.

(1) $$SN=\frac{TP}{\left(TP+FN\right)}\;\;\;\;\;\;\;\;\;SP=\frac{TN}{\left(TN+FP\right)}\;\;\;\;\;\;\;\;\;Gmean=\sqrt{SN\cdot SP}$$

### 2.4. Machine-Learning-Based Prediction Models

We propose the use of supervised learning to deal with this bi-class classification problem. The data classification is performed by a two-step process, consisting of learning and a classification phase. Thus, given a dataset *S* defined as in Equation (2), the objective of a classifier is to discover how the values of a vector containing different features are related with its associated label. Therefore, for a given set with *T* examples of the form $\left\{\left(x_{1},y_{1}\right),\ldots ,\left(x_{T},y_{T}\right)\right\}$, ML techniques seek to map the unknown relation between $x_{i}$ and $y_{i}$ by function f(x), where f:X→Y, with *X* representing the feature space and *Y* the output space.

(2) $$S=\left\{\left(x_{i},y_{i}\right)\right\},\;i=1,\ldots ,T$$

where $x_{i}$ is a sample in the *q*-dimensional feature space $x_{i}\in \left\{f_{1},f_{2},\ldots ,f_{q}\right\}$; $y_{i}$ is the class identity label $y_{i}\in \left\{0,1\right\}$; and *T* the total instances in the dataset. Instances refer to amount of nights available after the data processing that was used to make the predictions.

Personalized predictive models were generated using two supervised ML algorithms which have been widely applied in supervised learning: multilayer perceptron networks (MLP) [34] and support vector machines (SVM) [35]. As a result of the limited number of instances obtained for some patients, k-fold cross validation was applied in the entire dataset. This process should be repeated/iterated a number of times for problems with a small sample size [36]. An exhaustive feature selection method was applied and 2048 feature vectors were evaluated. For each combination of features, stratified random sampling was performed, and thus the balance was maintained between classes. Later, k-fold cross validation (k = 5) was conducted, and the results for that iteration were obtained. This procedure was repeated 100 times for each feature vector, and the final result was obtained by averaging the results from the 100 repetitions.

## 3. Results

A total of ten subjects were enrolled to participate in the study: 8 women, age 31.8 ± 16.8 years, HbA1c 7.3 ± 0.5%, body mass index 24.6 ± 3.6 kg/m^2^, and duration of diabetes 20.0 ± 8.9 years. All subjects completed the study. Table 1 presents a summary of CGM data for the whole period in which patients were monitored. The glucometrics, including coefficient of variation (CV) and time within and above different target glucose ranges, demonstrate that the participants enrolled in our study correspond to a high glycemic variability and hypoglycemia prone group of T1D patients.

The total number of instances generated after the data processing procedure and applied in the ML algorithms is presented in Table 2, as well as the distribution between instances labeled as Class 0 and Class 1. On average, nocturnal hypoglycemia occurred in one third of the nights that were used to make predictions. Differences in the number of instances available per subject are related to the fact that there were days without sleeping information provided by the Fitbit. This was probably due to patients having removed the Fitbit or the device not recording due to technical problems.

Table 3 presents the results for the ten patients, considering the feature vector that obtained the maximum Gmean among all the combinations of features evaluated, for both the MLP and SVM techniques. The averaged value over the 100 runs was considered for this purpose. These results confirm the feasibility of the proposed approach. The SVM achieved better outcomes for almost all the patients when compared with the MLP, mainly in the sensitivity analysis. Considering the median outcomes for the entire cohort, almost 80% of the nights with hypoglycemia would be avoided using this algorithm, while at the same time achieving more than an 80% specificity. P51 achieved an accuracy of 100%. However, the small number of instances obtained from this patient mean it is necessary to be skeptical as regards making further conclusions. The worst outcomes were obtained for P12 with the SVM models, with a Gmean of 64.31%.

Analyzing the outcomes achieved by the MLP for the entire cohort, the median sensitivity indicates that almost 70% of the nocturnal hypoglycemic events could be avoided, and in only 21% of the cases, patients would have taken an unnecessarily action due to a false positive prediction. Similarly, P12 achieved the worst outcomes with a Gmean of 63.28%, but even so, the results are acceptable since more than half of the hypoglycemia could be avoided.

## 4. Discussion

In this study, we assessed the feasibility of applying machine-learning-based prediction models to forecast the occurrence of hypoglycemia in the period when patients were sleeping (informed by a physical activity tracker). Our results suggest that this prediction model seems to be helpful to anticipate nocturnal hypoglycemia in T1D patients on MDI, using CGM and a physical activity tracker during challenging, real-life situations.

Although our methodology to predict NH was not embedded into a decision support system, the effects resulting from the use of our approach in clinical practice can be estimated. For instance, P56 generated a total of 55 instances with 20 hypoglycemia events. Considering the results obtained by the MLP approach, the system would be able to predict NH in 16 of these nights, failing in the prediction of the remaining four hypoglycemia events. In case of the prediction of hypoglycemia, the recommendation would be to eat a bedtime snack containing complex CH [37]. On the other hand, on the 35 nights when hypoglycemia was not observed in the instances set, in only three nights, patients would be alerted wrongly, with the consequence of an unnecessary CH snack being consumed.

In our work, the metric Gmean was adopted to select the best prediction model. This choice was motivated because it applies the same weight to the *SN* and *SP* metrics. Although the main objective is hypoglycemia avoidance, benefits should be balanced against potential side effects. The occurrence of excessive false positives may lead patients to hyperglycemia during the night. In addition, patients may gain weight due to excessive and unnecessary CH consumption at bedtime. Furthermore, better customization of the models could be achieved depending on the physiological response of patients for such corrective actions during the nighttime period.

An advantage of the approach presented in this work is the inclusion of features related with physical activity as input for the prediction models. It has already been shown that daytime physical activity is strongly related with NH [38,39,40]; however, previous investigations intended for NH prediction did not consider such information [28,41]. In our proposal, physical activity data were collected by the wristband. Such devices are able to provide an acceptable accuracy for our application. Moreover, they have been available for some time and are not a major burden for a patient to use in their daily life [42].

Our study has some limitations. Unfortunately, some patients presented a reduced number of instances. The data acquisition procedure was highly depended on the patients’ commitment to provide the information related to insulin dosing, CH intake, wearing the wristband, and performing sensor scans regularly. Regarding missing values of fast-acting insulin injections, which should have been saved manually by the patients, the possibility of using smart insulin pens in the forthcoming clinical trials intended to collect data from patients using MDI could help to reduce the influence of patients’ engagement on the quality of the data. A downside of the approach considered to predict NH in this work is the exhaustive feature selection methodology applied. A major limitation of this method is the computational cost required to evaluate all the feature vectors generated. For future applications, considering online training of predictive models, other feature selection techniques should be considered or even some dimensionality reduction techniques. The selection of the random forest technique could reduce the burden caused by the evaluation of different feature vectors, since the algorithm is based on random input variable selection and bootstrapping. Our study included a group of T1D patients particularly predisposed to NH. As a consequence, we do not know whether the results would apply to participants with a lower risk of hypoglycemia. Finally, it is necessary to embed the machine-learning-based predictions models into a decision support system to allow patients to make use of the predictions, and thus evaluate the efficacy of such an approach in real-life conditions. Future clinical trials are being prepared by our research group and should be conducted soon.

## 5. Conclusions

We propose a novel methodology intended to predict NH, mainly when patients are asleep. While NH is a major concern in T1D management, and physical activity is directly related with its incidence, very little work has been performed to aid patients to predict NH. In a recent publication by Jensen et al. using information from a database from a clinical trial performed in T1D using CSII, the authors claimed that by using machine-learning methods (linear discriminant function), it was possible to predict nocturnal hypoglycemia. However, they pointed out the limitation of the lack of physical activity measures in their study, as this information could improve the predictions [43]. Moreover, our study considered a group of patients under MDI therapy, which is frequently associated with NH, and the most frequently used type of insulin therapy for T1D worldwide. The results obtained are promising and encouraging, indicating that classical ML algorithms are suitable to deal with this problem using CGM and a physical activity tracker. Adoption of CGM has been growing rapidly over the past years and wearable activity trackers are already available. Future utilization of these machine-learning-based prediction models could be implemented in patients’ cell phones, based on a decision support system application. Therefore, patients would inform the system that they are preparing to sleep, and the decision system would advise the subject whether the consumption of a bedtime snack or other preventive maneuvers are deemed necessary.

In conclusion, this work supports the feasibility of using ML techniques to address the prediction of nocturnal hypoglycemia in the daily life of patients with T1D on MDI, using CGM and a physical activity tracker. Every case of anticipated hypoglycemia could be followed by preventive strategies, this certainly warrants further investigation. 

## Figures and Tables

**Table 1:** Summaryof continuous glucose monitoring (CGM) data (mg/dL) from each participant.

	Mean CGM	STD CGM	%CV CGM	70–180	<70	<54	>180	>250
**P12**	187.13	87.19	46.59	45.34%	6.15%	2.13%	48.51%	23.45%
**P18**	147.01	58.58	39.84	62.16%	9.90%	4.62%	27.95%	5.38%
**P23**	158.43	69.62	43.94	56.30%	8.82%	3.28%	34.88%	11.31%
**P29**	171.59	78.15	45.54	49.19%	8.20%	3.71%	42.61%	16.92%
**P34**	163.16	65.60	40.21	55.22%	5.74%	1.35%	39.03%	10.20%
**P40**	183.93	80.63	43.84	42.06%	7.91%	4.51%	50.03%	21.11%
**P45**	154.55	75.83	49.06	50.06%	14.45%	7.12%	35.49%	11.33%
**P51**	155.25	73.00	47.02	56.37%	10.81%	3.92%	32.82%	12.22%
**P56**	175.17	79.76	45.53	46.45%	8.68%	3.71%	44.87%	17.98%
**P62**	160.44	90.53	56.42	47.21%	16.30%	9.92%	36.49%	16.61%
**Median**	161.80	76.99	45.54	49.62%	8.75%	3.81%	37.76%	14.41%

**Table 2:** Total number of instances for ten patients enrolled in the study. Class 0 is defined as a sleep period without hypoglycemia and Class 1 is defined as a sleep period with hypoglycemia.

Patient ID	Total Instances	Class 1	Class 0
P12	104	24 (23%)	80 (77%)
P18	61	11 (18%)	50 (82%)
P23	78	15 (19%)	63 (81%)
P29	78	34 (44%)	44 (56%)
P34	73	13 (18%)	60 (82%)
P40	34	7 (21%)	27 (79%)
P45	34	21 (62%)	13 (38%)
P51	14	3 (21%)	11 (79%)
P56	55	20 (36%)	35 (64%)
P62	91	51 (56%)	40 (44%)

**Table 3:** Averaged sensitivity (*SN*), specificity (*SP*), accuracy, and Gmean for the best feature vector of each patient over the 100 repetitions performed for both multilayer perceptron networks (MLP) and support vector machines (SVM). Results are presented as a percentage.

Patient ID		Sensitivity			Specificity			Accuracy			*G_mean_*	
		MLP	SVM		MLP	SVM		MLP	SVM		MLP	SVM
P12		64.08	67.63		63.19	61.30		63.39	62.76		63.28	64.31
P18		65.45	76.91		79.10	71.98		76.64	72.87		71.73	74.34
P23		66.47	74.93		78.86	81.75		76.47	80.44		72.11	78.18
P29		65.71	78.35		72.98	82.20		69.81	80.53		69.00	80.20
P34		63.08	69.00		86.57	84.50		82.38	81.74		73.70	76.14
P40		74.43	80.57		88.22	88.74		85.38	87.06		80.69	84.37
P45		77.81	81.43		78.62	86.54		78.12	83.38		77.95	83.86
P51		100.00	100.00		99.73	100.00		99.79	100.00		99.86	100.00
P56		79.90	79.15		91.37	82.09		87.20	81.02		85.44	80.54
P62		72.57	79.53		70.15	75.97		71.51	77.97		71.19	77.70
Median		69.52	78.75		78.98	82.15		77.38	80.77		72.90	79.19

## Abbreviations

The following abbreviations are used in this manuscript:

NH	Nocturnal Hypoglycemia
MDI	Multiple Doses of Insulin
T1D	Type 1 Diabetes
CGM	Continuous Glucose Monitoring
CSII	Continuous Subcutaneous Insulin Infusion
MLP	Multilayer Perceptron
SVM	Support Vector Machine
IAH	Impaired Awareness Hypoglycemic
SAP	Sensor Augmented Pump
ML	Machine Learning
CH	Carbohydrate
SMBG	Self-Monitoring Blood Glucose
FSL	FreeStyle Libre
IOB	Insulin on Board
COB	Carbohydrate on Board
AOB	Activity on Board
